# Antifungal Activity of Amphiphilic Perylene Bisimides

**DOI:** 10.3390/molecules27206890

**Published:** 2022-10-14

**Authors:** Vicky C. Roa-Linares, Ana C. Mesa-Arango, Ramón J. Zaragozá, Miguel A. González-Cardenete

**Affiliations:** 1 Group of Dermatological Research and Group of Clinical Epidemiology, Instituto de Investigaciones Médicas, Universidad de Antioquia, Medellín 050010, Colombia; 2 Departamento de Química Orgánica, Universidad de Valencia, Dr. Moliner 50, 46100 Valencia, Spain; 3 Instituto de Tecnología Química, Universitat Politècnica de València-Consejo Superior de Investigaciones Científicas, Avda. de los Naranjos s/n, 46022 Valencia, Spain

**Keywords:** antifungal agents, perylene bisimide, *Candida* spp., *Fusarium* spp., *Sporothrix* spp., *Cryptococcus* spp., *Neoscytalidium* spp.

## Abstract

Perylene-based compounds, either naturally occurring or synthetic, have shown interesting biological activities. In this study, we report on the broad-spectrum antifungal properties of two lead amphiphilic perylene bisimides, compounds **4** and **5**, which were synthesized from perylene-3,4,9,10-tetracarboxylic dianhydride by condensation with spermine and an ammonium salt formation. The antifungal activity was evaluated using a collection of fungal strains and clinical isolates from patients with onychomycosis or sporotrichosis. Both molecules displayed an interesting antifungal profile with MIC values in the range of 2–25 μM, being as active as several reference drugs, even more potent in some particular strains. The ammonium trifluoroacetate salt **5** showed the highest activity with a MIC value of 2.1 μM for all tested *Candida* spp., two *Cryptococcus* spp., two *Fusarium* spp., and one *Neoscytalidium* spp. strain. Therefore, these amphiphilic molecules with the perylene moiety and cationic ammonium side chains represent important structural features for the development of novel antifungals.

## 1. Introduction

Onychomycosis is a chronic fungal nail infection, it accounts for 30% of superficial mycoses in dermatological consultations and up to 50% of all onychopathies, being particularly common in tropical countries [1]. The infection can affect the nail plate of one or more nails and cause discoloration, hyperkeratosis and/or a change in texture. This infection is a common cause of dermatological consultation as a result of its psychological, social and occupational impacts, being far more than a cosmetic problem [2]. In immunocompromised people, onychomycosis can have a more aggressive effect and can be the gateway for a disseminated infection [3]. Dermatophytes mainly of the genera *Trichophyton*, *Microsporum* and *Epidermophyton*, as well as the *Candida* species are the most common causative agents of onychomycosis [4]. However, non-dermatophyte filamentous fungi (NDF), such as species of *Fusarium*, *Aspergillus*, *Penicillium*, *Scopulariopsis*, *Scedosporium* and *Neoscytalidium* (before named *Scytalidium*) have increased significantly as the cause of onychomycosis, mainly in tropical zones [1]. Some *Fusarium* species are important not only for producing the nail infection, but also because they can cause invasive infections in immunocompromised patients [5]. Disseminated fusariosis is the most challenging and life-threatening manifestation with an estimated mortality rate between 66% and 75% [6]. The antifungal susceptibility of *Fusarium* spp. is variable and of relative resistance to most antifungal agents, some species are multiresistant [7].

Subcutaneous mycoses correspond to another important group of infections frequent in tropical and subtropical regions. They are characterized by a heterogeneous group of infections that often result from direct penetration of the fungus into the dermis and subcutaneous tissue through traumatic injury [8]. Sporotrichosis is the most frequent and worldwide distributed subcutaneous mycosis and is caused by members of the *Sporothrix schenckii* complex [9]. Despite advances in the development of antifungal agents in recent decades, the number of available molecules is still limited. In addition, a recently observed phenomenon in fungal infections is the increase in treatment failures brought on by drug interactions, potential liver toxicity and/or resistance of the causative agents. Therefore, new antifungal compounds with broader spectra and higher therapeutic indices are needed [4,10].

In the search for new therapeutic alternatives, natural products have received much attention as a source of novel antifungals [11]. Perylene-based compounds have been used in a variety of industrial applications for decades, especially in dye-sensitized solar cells, organic light-emitting diodes and organic thin film transistors. Moreover, these compounds have shown some anticancer, antiviral, antidepressant, and antimicrobial activity [12].

**Figure 1:**
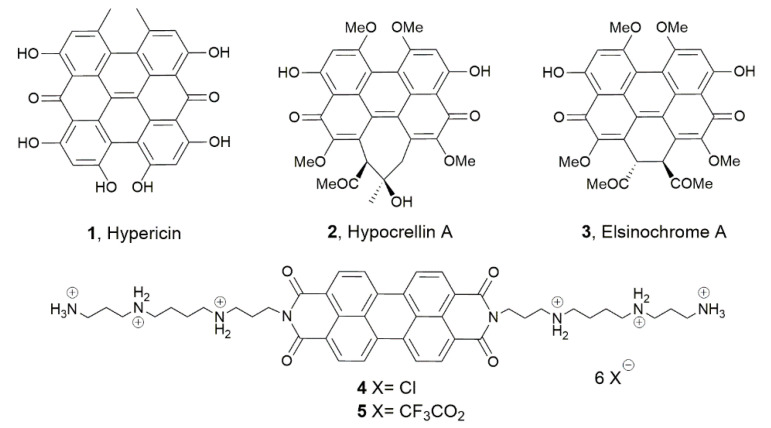
Examples of antifungal perylene-based compounds **1**–**3** and tested molecules **4** and **5**.

Naturally occurring perylene compounds, such as hypericin (**1**) and other perylene quinones, such as hypocrellin A (**2**) and elsinochrome A (**3**) (Figure 1), have shown some potential as antifungal agents [13,14,15]. They most likely act through a unique reactive oxygen species (ROS)-based mode of action, which results in a broad fungus-inhibiting spectrum as it occurs with amphotericin B (AmB) [13,16]. To date, however, the antifungal activity of readily available synthetic perylene derivatives has not been explored.

In a preliminary study carried out in our group [17], it was found that out of nine perylene bisimide derivatives, three displayed relevant antifungal in vitro activity against a reduced number of some clinically important yeast and filamentous fungi, **4** and **5** being the most active compounds. The other derivatives containing lipophilic side chains were inactive. The side chains were composed of diverse alkyl moieties and only compounds with chains formed with quaternary ammonium salts were active. Then, we decided to study these two molecules against a high number of strains of different fungi, some of them with recognized low susceptibility to traditional antifungals such as the *Fusarium* and *Neoscytalidium* species [18].

Herein, we describe the antifungal activity of two lead perylene bisimides containing cationic spermine side chains (**4**–**5**) (Figure 1) against a collection of fungal strains and clinical isolates from patients with onychomycosis or sporotrichosis, which included several *Candida* spp., *Fusarium* spp., and *Sporothrix* spp. These amphiphilic molecules were envisaged as antifungals since they could have a dual action by modifying the cell surface (as some cationic lipids do [19]) and/or the release of free radicals that impair the membrane’s permeability (as perylene quinones and amphotericin B do [13,16]).

## 2. Results

### 2.1. Chemistry

**Scheme 1:**
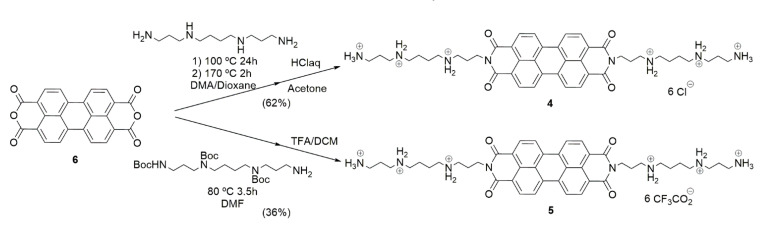
Synthesis of the tested compounds **4** and **5**.

Compounds **4** and **5** were synthesized starting from perylene-3,4,9,10-tetracarboxylic dianhydride (**6**) by condensation with the corresponding amine following the reported procedures [20,21], as shown in Scheme 1. Thus, perylene bisimide **4** was obtained by condensation of dianhydride **6** with excess spermine in two heating steps, followed by acidification with HCl and precipitation with acetone (62% yield). Similar thermal condensation with threefold tert-butoxycarbonyl-protected (Boc-protected) spermine followed by treatment with trifluoroacetic acid (TFA) afforded the corresponding perylene bisimide derivative **5** in a reasonable yield (36%).

### 2.2. Antifungal Activity

Compounds **4** and **5** (Scheme 1) were evaluated in vitro for antifungal activity against a collection of fungal strains and isolates of yeasts and filamentous fungi from patients with onychomycosis or sporotrichosis (47 strains), including several *Candida* spp., *Fusarium* spp., *Neoscytalidium* spp., *Cryptococcus* spp., and *Sporothrix* spp., as well as for cytotoxicity on Vero cells following established procedures (see Methods section). The results expressed as the minimal inhibitory concentration values (MICs) for antifungal activity are summarized in Table 1, while the inhibitory concentration 50% (IC_50_) for cytotoxicity and selectivity indices are displayed in Table 2 and Table 3, respectively.

As can be seen in Table 1, the most susceptible strains were *Candida* spp. and the two species of *Cryptococcus* with MIC values of 6.4 and 2.1 µM for compounds **4** and **5**, respectively.

In addition, both molecules showed low MIC values against the filamentous fungi *Scedosporium* spp., *Neoscytalidium* spp., *Sporothrix* spp., and *Trichophyton mentagrophytes* ATCC 24198 (also known as *T. interdigitale*). The MIC values for compound **4** were between 6.4 and 12.8 µM, while for compound **5** the MICs range was 2.1–4.3 µM. The two species of *Aspergillus* were, however, the least susceptible to the perylene derivatives with MICs ≥ 17.3 µM. The susceptibility of the clinical isolates of *Fusarium* spp. to the perylene bisimide derivatives was variable. For example, of the seventeen clinical isolates of *Fusarium* spp., compound **4** showed low MIC values (6.4 µM) in two isolates, a MIC value of 12.8 µM in another, and the remaining showed MIC values ≥ 25.6 µM. On the other hand, compound **5** showed low MICs (2.1–8.6 µM) in seven strains. In addition, three of the *Fusarium* strains that were sensitive to **4** were also sensitive to **5** (MIC ranges 6.4–12.8 and 2.1–4.3 µM, respectively). *Fusarium oxysporum* ATCC 48112 was more susceptible to our derivatives than most of the clinical isolates (MIC values of 9.6 and 4.3 µM for **4** and **5**, respectively).

In a parallel manner to the evaluation of synthetic molecules, the MICs of three of the most commonly used antifungals in clinical practice were determined with some reference strains and clinical isolates as controls of the technique. Itraconazole was evaluated with all *Sporothrix* spp. isolates (10). Only two of them showed high MIC values (≥22.7 µM), but the others showed low MIC values (≤1.4 µM). Conversely, *Neoscytalidium* spp., and *Fusarium* spp. were less susceptible to itraconazole, showing high MIC values (≥22.7 µM); two isolates of *Fusarium* spp. were the exception with a low MIC (2.8 µM). Itraconazole MICs with the reference strains were within the range defined by the ASFT-EUCAST protocol (0.18 µM or 0.125 µg/mL for *A. flavus* ATCC 204304, and 0.35 µM or 0.25 µg/mL for both *C. krusei* ATCC 6258 and *A. fumigatus* ATCC 204305). Terbinafine is one of the most important antifungals in the treatment of onychomycosis [22]. It was observed that the in vitro susceptibility of *Fusarium* and *Neoscytalidium* species was variable (MIC ranges were 3.4–≥13.7 µM and 0.9–≥13.7 µM, respectively). Nevertheless, *T. mentagrophytes* ATCC 24198 was highly susceptible to this allylamine (MIC value ≤ 0.03 µM). Additionally, the MIC values of amphotericin B for *A. fumigatus* ATCC 204305 and *A. flavus* ATCC 204304 (1 µg/mL or 1.08 µM and 0.5 µg/mL or 0.54 µM, respectively) were within the accepted range of the technique.

In order to determine the cytotoxicity of the perylene bisimide derivatives, the viability of normal mammalian Vero cells treated with both compounds was evaluated and the corresponding inhibitory concentration of 50% (IC_50_) and the selectivity indices (SI: IC_50_ of Vero cells/MIC of each fungus) were calculated (see Table 2 and Table 3). IC_50_ and SI values were calculated at three different times (24, 48 and 72 h), considering the incubation time for each fungal strain in the antifungal activity assay. In all cases, compounds **4** and **5** showed SI values greater than 1, except against five strains of *Fusarium* spp. (SI = 0.7). Importantly, compound **5** showed the best selectivity against all the tested fungal strains (SI in the range of 2.8–5.6) in comparison with the SI values obtained for compound **4** (see Table 3), except against *Candida* spp. (5.9), confirming the relevant and selective antifungal activity of this compound against some clinically important pathogens.

## 3. Discussion

At present, the treatment options of fungal infections are restricted to only four chemical classes of antifungals represented by polyenes, azoles, flucytosine, and echinocandins, which have good performance, but based on the number of affected individuals and the number of deaths from candidiasis, aspergillosis, and cryptococcal meningitis, among other diseases, it is clear that the search for new antifungal molecules is a pending task [23]. The results show that both perylene bisimide derivatives, **4** and **5**, have broad-spectrum antifungal activity, making compound **5** the most active. Although both molecules are ammonium derivatives and are structurally similar, it is possible that the higher antifungal activity of compound **5** can be attributed to the difference in the positive charge densities of the side chains, which leads to a disruption of the microbial cell surface [24].

**Figure 2:**
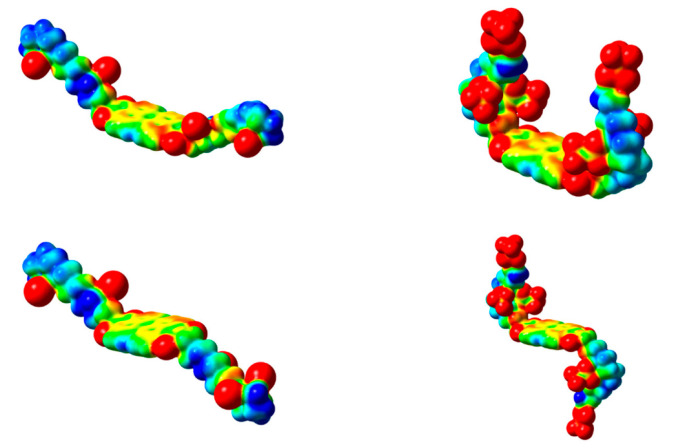
Electron density cloud for the hydrochloride **4** (**left**) and trifluoroacetate salt **5** (**right**) in CIS (**up**) and TRANS (**down**) dispositions of side chains.

**Figure 3:**
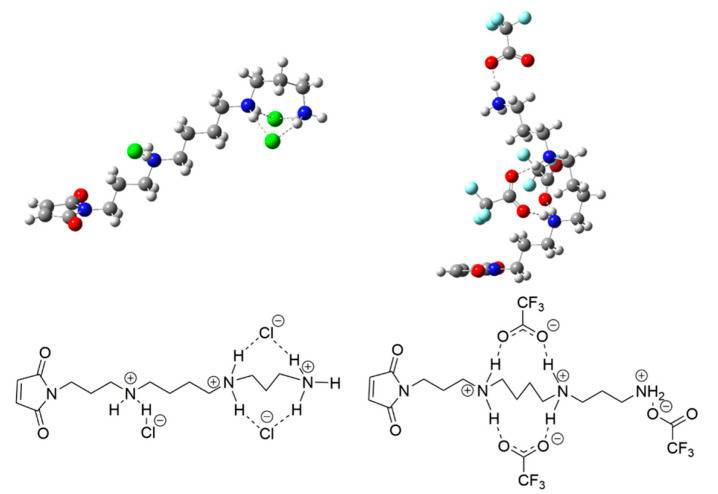
Energetically favored conformations for side chain models of the hydrochloride **4** (**left**) and trifluoroacetate salt **5** (**right**).

**Figure 4:**
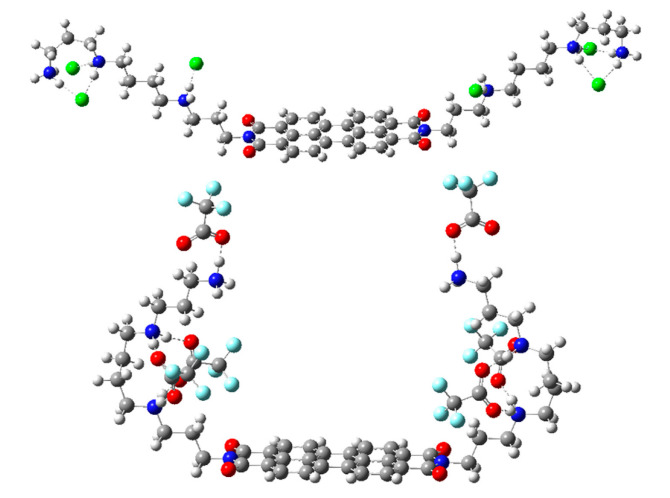
Energetically favored conformations for the hydrochloride **4** (**up**) and trifluoroacetate salt **5** (**down**).

Molecular modelling after side-chain conformational analysis indicates differences in total surface charge densities with CHELPG charge distributions of + 3.99 and + 4.22 for the polycationic nuclei of **4** and **5** in both cis and trans dispositions, respectively (see Supplementary Materials and Figure 2). More importantly, a dramatic change in conformation with both counter ions was found (see Supplementary Materials and Figure 3 and Figure 4). Chloride ions prefer an elongated conformation of the side chain, which resides slightly above and below the perylene plane. Trifluoroacetate ions cause a dramatic change since the chains are orthogonal to the perylene plane. These differences in the electronics between compounds **4** and **5** and above all the change in shape might be responsible for their different bioactivity profiles. Other authors have also found bioactivity differences between trifluoroacetate and hydrochloride salts of cationic amphiphilic drugs [25].

The anti-*Candida* activity of these molecules is quite relevant because in recent years a significant increase in resistant strains has been detected, as well as strains with low sensitivity or with the ability to develop resistance [26]. The activity of the perylene bisimide derivatives against the *Cryptococcus* species is also interesting, considering that these yeasts are resistant to echinocandins and their resistance to fluconazole is rising. This antifungal is one of the most important in the treatment of cryptococcosis [23].

The activity of molecules **4** and **5** against *Neoscytalidium* spp. is also worth noting (see Table 1). Fungi belonging to the genus *Neoscytalidium* spp. are phytopathogens widely distributed around the world. However, some species are involved in human pathology. These fungi are an important cause of onychomycosis, mainly in tropical countries, and the treatment response is poor [27]. Itraconazole showed high MIC values (≥22.7 µM) with *Neoscytalidium* spp. and terbinafine (ranged between 0.8 and ≥13.7 µM). In addition, some *Fusarium* species are phytopathogens, but they have the ability to cause human infections such as onychomycosis, hyalohifomicosis, keratitis and disseminated diseases associated with high mortality. The most pathogenic species for humans are *F. solani* and *F. oxysporum*, unfortunately, the sensitivity of *Fusarium* spp. to the main antifungals is variable [6,7,28]. Contrary to what was observed with *Neoscytalidium* spp., *Fusarium* spp. were less susceptible to the perylene bisimide derivatives. Minimal Inhibitory Concentrations in 78% and 67% of *Fusarium* spp. isolates were ≥25.6 µM and ≥17.3 µM for the molecules **4** and **5**, respectively. The low or variable susceptibility situation, and its importance in both human and plant infections, highlights the need for new active molecules against this group of fungi.

The study of the mechanism of action of these molecules is an important topic since apparently it is different from that of the azoles and the amphotericin B (independent of the route of the ergosterol biosynthesis or the interaction with ergosterol). This could be proposed based on the behavior observed with some fungi. For example, *C. tropicalis* ATCC 200956 is resistant to azoles and amphotericin B [29], however, it was susceptible to the two perylene bisimide derivatives, with MIC values of 2.1 and 6.4 µM for the molecules **5** and **4**, respectively, as well as the other species of *Candida* that are also susceptible to azoles and to amphotericin B. Moreover, the fact that the *Cryptococcus* spp. strains have been susceptible to the two molecules, suggests that the target of echinocandins is not the same since this genus is resistant to this antifungal [30]. Previous work has shown that perylene derivatives have antitumor activity because they are pro-oxidants that facilitate or induce apoptosis and necrosis in a wide spectrum of cancer cell types [31]. In addition, it has been demonstrated that amphotericin B induces oxidative stress [16]. Therefore, it is possible that the mechanism of action of these molecules is also related to oxidative stress.

## 4. Materials and Methods

### 4.1. Chemistry

Compounds **4** and **5** have been prepared following similar conditions of the reported procedures [20,21]. All compounds prepared in this work exhibit spectroscopic data in agreement with the proposed structures and reported data. The purity of the final compounds was estimated at around 93–95% for both compounds determined by elemental analysis, considering water as the main impurity. It was found that both compounds did not burn well, especially the fluor-containing bisimide, but further experiments to optimize the analysis were not conducted due to the aggressiveness of fluorine for the analyzer.

#### 4.1.1. Synthesis of Compound **4**

Adapted from Franceschin et al. [20], Perylene-3,4,9,10-tetracarboxylic dianhydride (140 mg, 0.35 mmol) was mixed with an excess of the solid spermine (1.6 g, 8 mmol) in a mixture of dimethylacetamide (4 mL) and dioxane (4 mL). The mixture was heated at 100 °C for 24 h and later at 170 °C for 2 h. Then, it was cooled to rt and a mixture of diethyl ether/1-propanol (4:1, 10 mL) was added. The resulting precipitate was filtered under vacuum and dissolved with 20 mL of 6 M HCl and 20 mL of concentrated HCl and 70 mL of acetone was added. The resulting precipitate was filtered off, washed with acetone and dried under vacuum to give 210 mg (62% yield) of a dark red solid. ^1^H NMR (300 MHz, CF3COOD) *δ* 9.31 (8H, m, aromatic H), 7.92 (4H, s), 4.97 (4H, s, N_imidic_-CH_2_, signal taken as reference to compare with literature values^1^), 3.85 (20H, m), 2.88 (8H, m), 2.48 (8H, m); ^13^C NMR (75 MHz, CF3COOD) *δ*_C_ 161.7 (s), 131.8 (s), 128.6 (d), 124.8 (s), 121.9 (s), 119.8 (d), 117.2 (s), 43.7 (t), 43.5 (t), 41.8 (t), 41.1 (t), 33.3 (t), 32.8 (t), 19.9 (t), 19.2 (t), 18.4 (t); Elemental analysis: C_44_H_56_N_8_O_4_ (6HCl): calcd C: 53.9%, N: 11.4%, H: 6.3%; found C: 53.5%, N: 10.7%, H: 6.2%.

#### 4.1.2. Synthesis of Compound **5**

Adapted from Würthner et al. [21], Perylene-3,4,9,10-tetracarboxylic dianhydride (290 mg, 0.75 mmol) and the appropriate Boc-protected spermine (900 mg, 1.8 mmol), obtained from spermine and ethyl trifluoroacetate in methanol at −78 °C followed by treatment with di-tert-butyldicarbonate and removal of the trifluoroacetate group with aqueous NH_3_ [32], were suspended in dry DMF (6 mL) and stirred for 5 h at 80 °C. The reaction was cooled and diluted with H_2_O (20 mL) and DCM (140 mL). The organic phase was washed with H_2_O (3 × 70 mL). The aqueous phase was extracted with DCM (2 × 30 mL). The combined organic extracts were dried and concentrated to give a residue which was chromatographed on silica gel eluting with DCM-MeOH 97:3 to afford 450 mg of Boc-protected perylene bisimide. The latter (418 mg, 0.3 mmol) was dissolved in DCM (30 mL) and 30 mL of TFA was added, and the mixture was stirred at rt for 1 h. Then the solvent and TFA were removed in vacuo, giving a red solid which was dissolved in H_2_O (45 mL) and submitted to lyophilization. As a result, 360 mg (36% overall yield) of a red fluffy solid were obtained. ^1^H NMR (300 MHz, DMSO-d6) *δ* 8.80 (6H, br s), 8.48 (4H, d, *J* = 8), 8.24 (4H, br s), 4.14 (4H, br s, N_imidic_-CH_2_), 3.10–2.80 (20H, m), 2.04 (4H, br s), 1.90 (4H, m), 1.63 (8H, m); The integral of aromatic signals was not accurate due to the ammonium group protons being overlapped; ^13^C NMR (75 MHz, DMSO-d6) *δ*_C_ 162.7 (s), 158.5 (q, J_C-F_ = 30 Hz, CF_3_COO), 133.4 (s), 130.5 (d), 127.9 (s), 124.8 (s), 123.8 (d), 122.1 (s), 117.0 (q, J_C-F_ = 300 Hz, CF_3_COO), 46.1 (t), 46.0 (t), 44.8 (t), 43.8 (t), 37.2 (t), 36.2 (t), 24.5 (t), 23.7 (t), 22.7 (t), 22.6 (t); Elemental analysis: C_56_H_62_F_18_N_8_O_16_: calcd C: 46.5%, H: 4.3%, F: 23.7%, N: 7.7%, found C: 45.9%, N: 8.1%, H: 4.2%.

### 4.2. Bioassays

#### 4.2.1. Antifungal Activity

##### Micro-Organisms

Two perylene bisimide derivatives were evaluated with 47 fungal strains. Nine yeast strains of reference (*Candida glabrata* ATCC 90030, *C. tropicalis* ATCC 750, *C. tropicalis* ATCC 200956, *C. albicans* SC5314, *C. krusei* ATCC 6258, *C. lusitaniae* ATCC 2819, *C. albicans* ATCC 64548, *Cryptococcus gattii* CBS 10865, and *C. neoformans* var *grubii* ATCC 208821) were included. Furthermore, 37 filamentous fungi were distributed between the clinical isolations of *Fusarium* spp. (17), *Neoscytalidium* spp. (6), and *Scedosporium* spp. (1) from patients with onychomycoses. Ten clinical isolates of *Sporothrix* spp. from patients with sporothrichosis were evaluated. The following reference strains of filamentous fungi were included: *Fusarium oxysporum* ATCC 48112, *Aspergillus flavus* ATCC 204304, and *Aspergillus fumigatus* ATCC 204305 and *Trichophyton mentagrophytes* (*T. interdigitale*) ATCC 24198.

##### Antifungal Assay

The in vitro antifungal activity of two perylene bisimide derivatives was determined following the Antifungal Susceptibility Testing Subcommittee of the European Committee on Antibiotic Susceptibility Testing (AFST-EUCAST) reference procedure for yeasts (http://www.eucast.org/ast_of_fungi/methodsinantifungalsusceptibilitytesting/susceptibility_testing_of_yeasts/) and for filamentous (http://www.eucast.org/ast_of_fungi/methodsinantifungalsusceptibilitytesting/susceptibility_testing_of_moulds/). The antifungal tests were carried out in *Roswell Park Memorial Institute medium (RPMI)* buffered at pH = 7 with 3-Morpholinopropanesulfonic acid (MOPS). Compounds were evaluated at five final concentrations (25, 12.5, 6.25, 3.125, and 1.56 µg/mL). One hundred microliter dilutions 2X of each concentration were dispensed into 96-well microtitration plates and the same volumes of each inoculum at 1–5 × 10^5^ UFC/mL were added. Minimal Inhibitory Concentration (MIC) was defined as the lowest dilution that resulted in total inhibition of visible growth after incubation at 28 °C during 48 h in the case of filamentous fungi, except for *Sporothrix* spp. and *T. mentagrophytes* ATCC 24198, which were incubated for six days. Minimal Inhibitory Concentrations of *Candida* spp. were determined after 24 h of incubation at 37 °C, while *Cryptococcus* spp. were incubated 48 h at the same temperature. The positive control employed was itraconazole (Sigma-Aldrich, Co., St. Louis, MO, USA), at a concentration range of 16–1 µg/mL against *F. oxysporum* ATCC 48112, *C. krusei* ATCC 6258, *A. flavus* ATCC 204304 and *A. fumigatus* ATCC 204305. Terbinafine (Recalcine laboratories, Santiago de Chile, Chile) was evaluated at concentration ranges from 4 to 0.0078 µg/mL with T. mentagrophytes ATCC 24198. Finally, amphotericin B (Sigma-Aldrich, Co., MO, USA), was evaluated at a concentration range of 16 to 1 µg/mL with *A. flavus* ATCC 204304 and *A. fumigatus* ATCC 204305. Moreover, some clinical isolates were tested with these antifungals. The tests were performed in duplicate in two different assays.

#### 4.2.2. Cytotoxic Activity

##### Cell Culture

Vero cells (African green monkey kidney-*Cercopithecus aethiops*, ATCC: CCL 81 line). The cells were maintained in Dulbecco’s Modified Eagle Medium (DMEM) supplemented with 5% inactivated Fetal Bovine Serum (FBS), 100 units/mL of penicillin, 100 μg/mL of streptomycin, 100 μg/mL of l-glutamine, 0.14% NaHCO_3_, and 1% of each non-essential amino acid and a minimum essential medium vitamin solution (choline chloride, D-calcium pantothenate, folic acid, nicotinamide, pyridoxal hydrochloride, riboflavin, thiamine hydrochloride and i-inositol).

##### Cytotoxicity Assay

The cytotoxicity of perylene derivatives was determined using an MTT assay (dimethylthiazol-2-yl]-2,5-diphenyl tetrazoliumbromide) as described previously [33]. Briefly, Vero cells were seeded into 96-well plates (2.0 × 10^4^ cells/well) and incubated for 24 h at 37 °C. After incubation, each dilution of compounds (400 µg/mL–3.125 µg/mL) was added to the appropriate wells and the plates were incubated for an additional 24 h, 48 h or 72 h at 37 °C in a humidified 5% CO_2_ atmosphere. Subsequently, the culture medium was removed and 28 μL of MTT solution (2 mg/mL) prepared in DMEM was added and incubated for 1½ h at 37 °C in a humidified 5% CO_2_ atmosphere; then 130 μL of DMSO was added to dissolve the formazan crystals. Optical densities (OD) were read at 570 nm. The minimal dilution of compounds that induced a 50% growth inhibition of the cells was expressed as Inhibitory Concentration 50% (IC_50_). The IC50 values were obtained by linear regression analysis of the dose–response curves generated from the absorbance data using GraphPad Prism version 6.00 for Windows, GraphPad Software (La Jolla, CA, USA, www.graphpad.com), and were expressed as the Mean ± Standard Deviation (M ± SD) of two independent experiments performed in quadruplicate, except for the 72 h experiments, which have only one data point. To calculate the antifungal selectivity index (SI), the cytotoxicity in Vero cells and the MIC against each fungus was compared (SI = IC_50_/MIC).

### 4.3. Computational Methods

Optimization and CHELPG charges were carried out with the Gaussian 09 suite of programs [34] at the B3LYP/6-31G ** level [35]. The inclusion of solvent effects has been considered by using a relatively simple self-consistent reaction field (SCRF) method [36,37] based on the polarizable continuum model (PCM) of Tomasi’s group [38,39,40]. Geometries have been fully optimized with PCM. The solvent we have used was water (solvent used in biological assays). Surfaces of electron density were obtained with the GaussView 5 program (Gaussian, Inc., Wallingford, CT 06492 USA) using “electron density from Total SCF Density (isoval = 0.004; [mapped with ESP])”. The colors used in rendering the mapped surface are based on a scaling between minimum (−1.000 × 10^−5^) and maximum (1.000 × 10^−1^) values.

## 5. Conclusions

In summary, a study of antifungal activity in vitro of two lead perylene bisimide derivatives and their corresponding conformational disposition together with charge distribution has been carried out. Compounds **4** and **5** were found to be highly active at low micromolar concentrations. Our results indicate important selectivity of both compounds, mainly against *Candida* spp., *Cryptococcus* spp., *Fusarium* spp., *Neoscytalidium* spp. and *T. mentagrophytes* (*T. interdigitale*) with SI values higher than **5**. The selectivity index is an important indicator of bioactivity and helps in designing more potent compounds. In particular, the trifluoroacetate salt **5** has an important antifungal activity in vitro against an important number of different isolates of pathogenic fungi, suggesting that this molecule could be a candidate for further studies with a greater number of isolates, including strains resistant to different antifungals. Furthermore, a study of the toxicity on skin cell lines, the mechanism of action and the efficacy in animal models are topics to consider in future studies.

## Figures and Tables

**Table 1:** Antifungal activity (MICs, μM) of perylene bisimide derivatives and controls against different fungal strains.

Fungal Strain (Number Strains)			MICs (µM) ^a,b^		
	4	5	Itraconazole	AmpB	Terbinafine
*Aspergillus flavus* ATCC 204304 (1)	≥25.6 (1)	≥17.3 (1)	0.18	0.54	
*Aspergillus fumigatus* ATCC 204305 (1)	≥25.6 (1)	≥17.3 (1)	0.35	1.08	
*Candida* spp. (2), *C. glabrata*, *C. tropicalis*, *C. albicans*, *C. krusei*, *C. lusitaniae*	6.4 (7)	2.1 (7)	0.06–1.42 (7)	0.05–0.16 (7)	
*Cryptococcus* spp., *C. neoformans*, *C.gattii* (2)	6.4 (2)	2.1 (2)			
*Fusarium oxysporum* (5)	12.8 (1) ≥25.6 (4)	4.3 (1)			≥13.7 (1)
		8.6 (1)	≥22.7 (5)		6.9 (2)
		17.3 (3)			3.4 (2)
*Fusarium oxysporum* ATCC 48112 (1)	9.6 (1) ^c^	4.3 (1)	≥22.7		6.9
*Fusarium proliferatum* (1)	≥25.6 (1)	≥17.3 (1)	≥22.7		≥13.7
*Fusarium solani* (2)	≥25.6 (2)	4.3 (1)	≥22.7 (2)		6.9 (1)
		≥17.3 (1)			≥13.7 (1)
*Fusarium* spp. (9)	6.4 (2)	2.1 (2) 4.3 (1) ≥17.3 (6)	≥22.7 (7)		≥13.7 (7)
	≥25.6 (7)		2.8 (2)		6.9 (2)
*Scedosporium* spp. (1)	12.8 (1)	4.3 (1)			
*Neoscytalidium dimidiatum* (3)	12.8 (3)	4.3 (3)	≥22.7 (3)		≥13.7 (2)
					0.9 (1)
*Neoscytalidium* spp. (3)	12.8 (3)	2.1 (1)	≥22.7 (3)		≥13.7 (1)
		4.3 (2)			3.4 (2)
*Sporothrix* spp. (10)	12.8 (10)	4.3 (10)	≥22.7 (2)		
			≤1.4 (8)		
*Trichophyton mentagrophytes* ATCC 24198 (1)	6.4 (1)	4.3 (1)			≤ 0.03

^a^ MIC, values of minimal inhibitory concentration, defined as the lowest dilution that resulted in total inhibition of visible growth corresponding to the average of the values obtained in the different experiments. Duplicates in two different days gave same values. ^b^ In round brackets, the number of isolates are indicated. ^c^ Geometric mean of different values obtained in two days by duplicate (12.8 µM and 6.4 µM).

**Table 2:** Cytotoxicity (IC_50_ in µM) on Vero cells of perylene bisimide derivatives after 24, 48 and 72 h of incubation.

Compound	IC_50_^a^		
	24 h	48 h	72 h
**4**	37.6 ± 2.0	34.2 ± 0.8	32.6
**5**	10.9 ± 2.7	12.1 ± 2.0	19.4

^a^ Inhibitory concentration 50 defined as concentration of compound that induces 50% of growth inhibition. Mean ± standard deviation (M ± SD) of two independent experiments performed in quadruplicate, except for 72 h only one data point.

**Table 3:** Range of selectivity index (SI) values of compounds against fungal strains.

Fungal Strain	Time (h)	SI ^a^	
		4	5
*Candida* spp.	24	5.9	5.1
*Aspergillus* spp.	48	1.3	0.7
*Cryptococcus* spp.		5.3	5.6
*Fusarium* spp.		2.7–5.3	0.7–5.6
*Scedosporium* spp.		2.7	2.8
*Scytalidium* spp.		2.7	2.8–5.6
*Sporothrix* spp.	72	2.5	4.5
*Trichophyton mentagrophytes*		5.1	4.5

^a^ SI, selectivity index defined as Vero IC_50_ over MIC.

## Data Availability

Not applicable.

## Supplementary Materials

The following supporting information can be downloaded at: https://www.mdpi.com/article/10.3390/molecules27206890/s1: Figures S1 and S2: Conformation diagrams of reduced model including side chains.; Figures S3 and S4. Conformation diagrams of perylene compounds **4** and **5**, CHELPG charges and electron density surfaces, and copies of ^1^H NMR and ^13^C NMR spectra for key synthesized compounds.

## Sample Availability

Samples of the compounds are not available from the authors.
