# Supplementary material for: Antifungal Activity of Amphiphilic Perylene Bisimides
Source: Molecules. 2022 Oct 14;27(20):6890. doi: 10.3390/molecules27206890 (PMC9609932; doi:10.3390/molecules27206890)
Supplement: Supplementary file 1 [file molecules-27-06890-s001.zip › molecules-1952022-supplementary.pdf]

Supplementary Information

Antifungal activity of amphiphilic perylene bisimides

Vicky C. Roa-Linares[1], Ana C. Mesa-Arango[1,*] Ramón J. Zaragozá[2], and Miguel A. González-Cardenete[3,*]

[1] Group of Dermatological Research and Group of Clinical Epidemiology, Instituto de Investigaciones Médicas, Universidad de Antioquia, 050010 Medellín, Colombia.

[2] Departamento de Química Orgánica, Universidad de Valencia, Dr Moliner 50, 46100 Burjassot, Valencia, Spain

[3] Instituto de Tecnología Química, Universitat Politècnica de Valencia-Consejo Superior de Investigaciones Científicas, Avda de los Naranjos s/n, 46022 Valencia, Spain.

Contents

Conformational analysis: reduced model……………..…………………………....S2-5

Conformational analysis: cis and trans dispositions.…………………………....S6-7

CHELPG charges and electron density surfaces for bisimides **4** and **5** …...........S8-9

Copies of ^{1}H NMR and ^{13}C NMR Spectra for key compounds **4** and **5**:

-^{1}H NMR spectrum of **4**……………...……….……………………………………S10

-^{13}C NMR spectrum of **4**…………..…..……………………………………………S11

-DEPT135 spectrum of **4**………………………………………………………………S12

-^{1}H NMR spectrum of **5**……………...……….……………………………………S13

-^{13}C NMR spectrum of **5**…………..…..……………………………………………S14

-DEPT135 spectrum of **5**………………………………………………………………S15

Conformational analysis

With the aim of carrying out a detailed study of the polycationic nucleus' conformations of **4** and **5**, the following reduced model has been used:

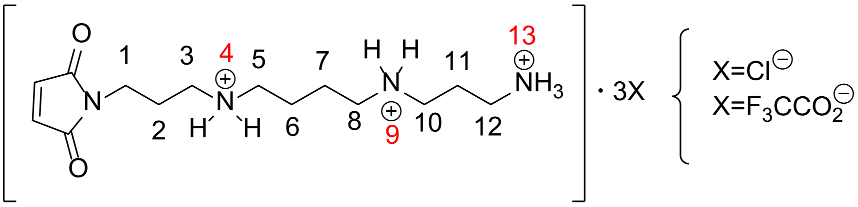

Figure S1. Conformation diagrams of reduced model including side chains. The conformers are shown at their calculated lowest energy conformation (ball and stick rendering).

X=Cl ⊖

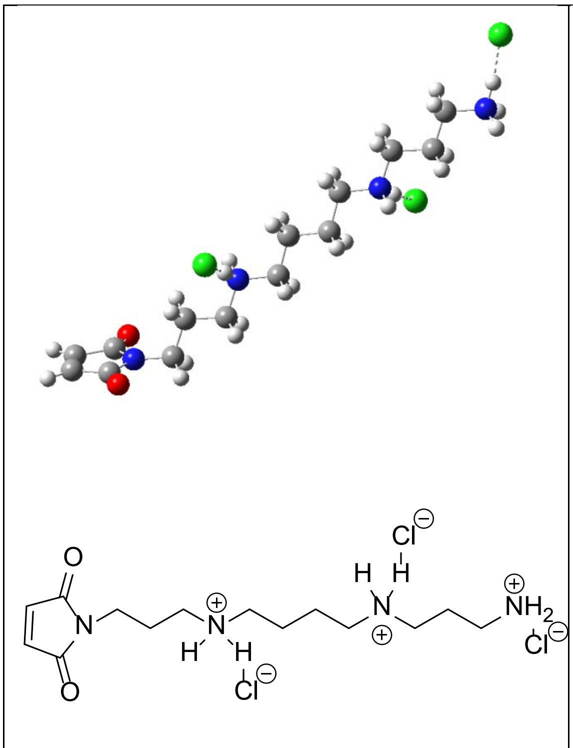

Figure S1-a

E= -2301.200479 (a.u.) ΔE= 7.3 Kcal/mol

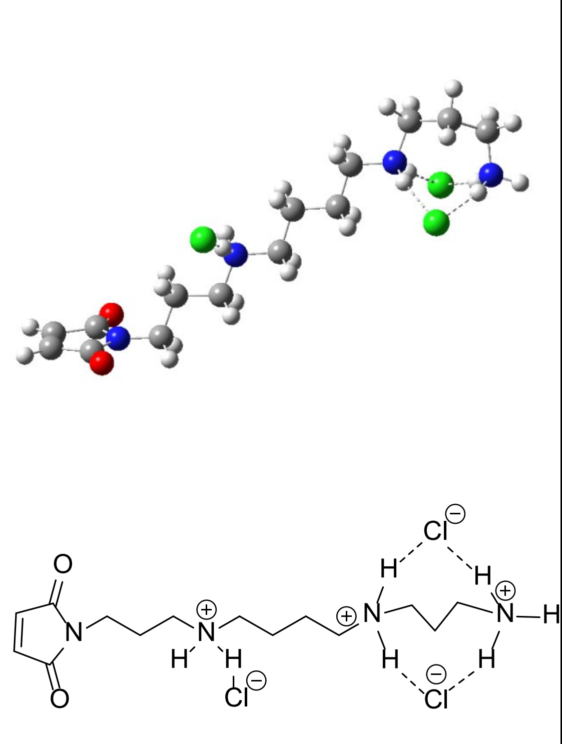

Figure S1-b

E=-2301.212184 (a.u.) ΔE= 0.0 Kcal/mol

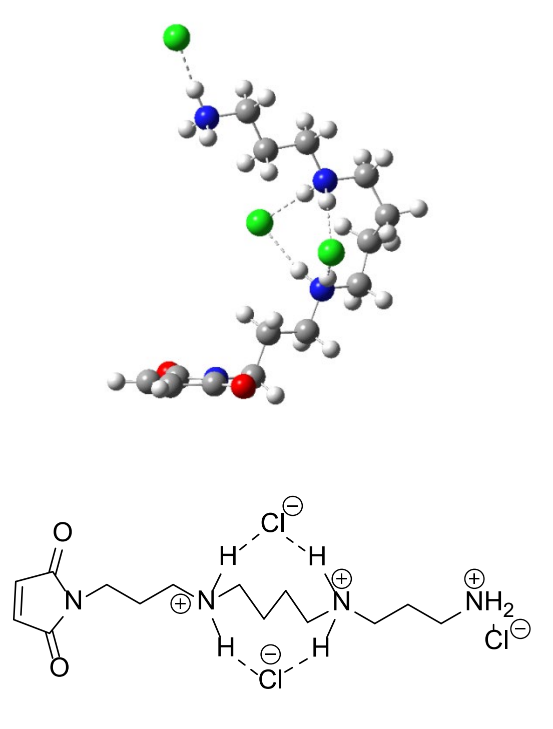

Figure S1-c
E=-2301.210538 (a.u.) ΔE= 1.0 Kcal/mol

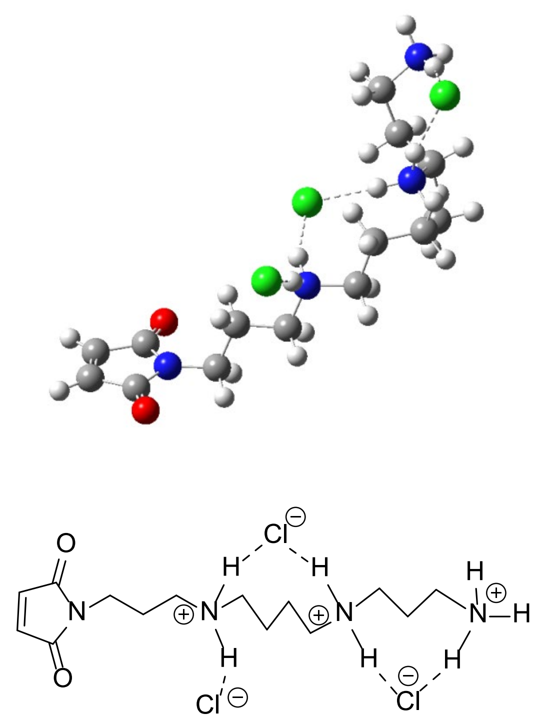

Figure S1-d
E=-2301.210406 (a.u.) ΔE= 1.1 Kcal/mol

Figure S2. Conformation diagrams of reduced model including side chains. The conformers are shown at their calculated lowest energy conformation (ball and stick rendering).

X=$F_3CCO_2^{\ominus}$

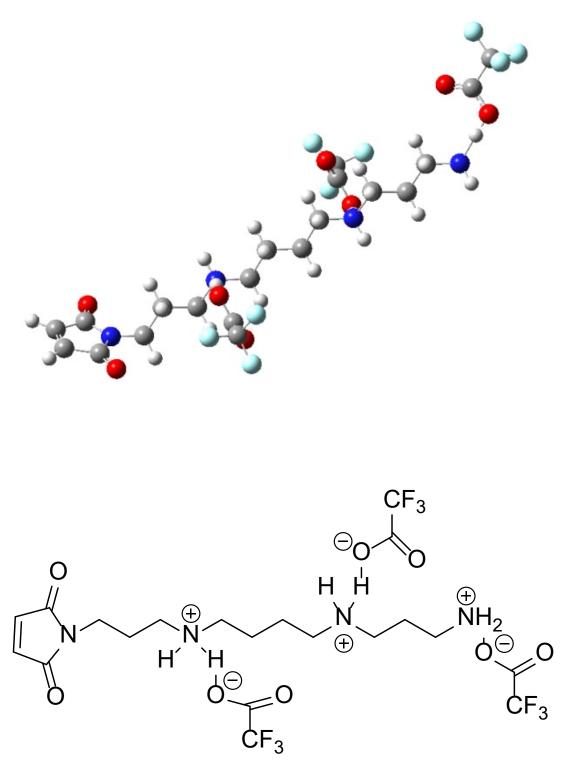

Figure S2-a
E=-2499.123316 (a.u.) ΔE= 13.9 Kcal/mol

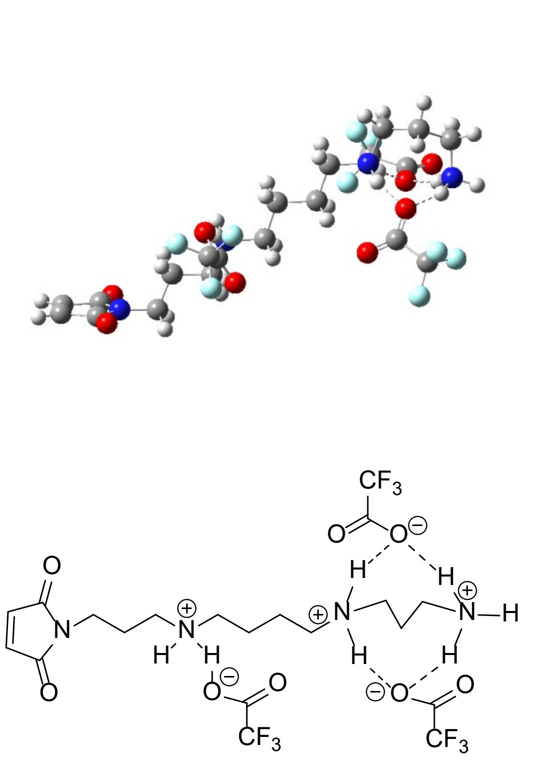

Figure S2-b
E=-2499.137814(a.u.) ΔE= 4.9 Kcal/mol

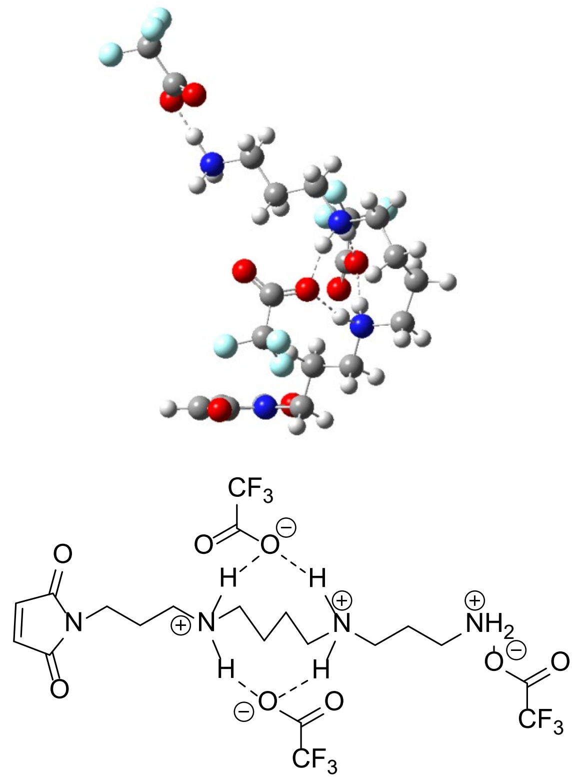

Figure S2-c
E=-2499.137930 (a.u.) ΔE= 4.8 Kcal/mol

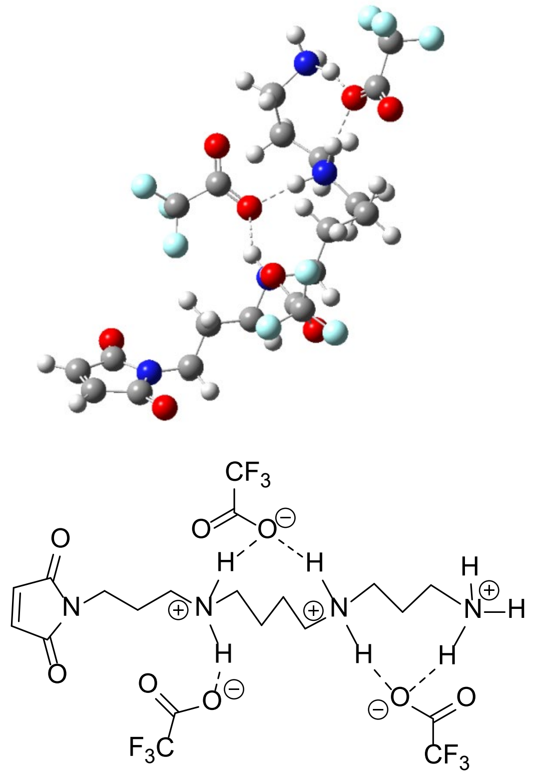

Figure S2-d
E=-2499.134533 (a.u.) ΔE= 6.9 Kcal/mol

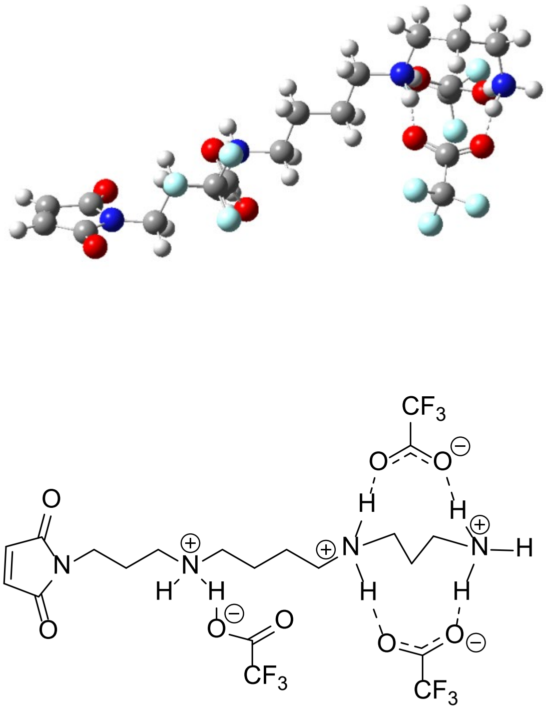

Figure S2-e
E=-2499.141360 (a.u.) ΔE= 2.6 Kcal/mol

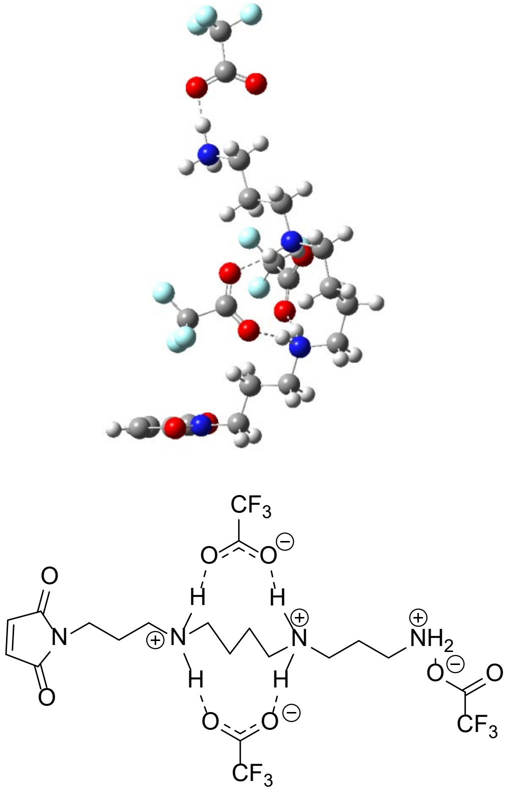

Figure S2-f
E=-2499.145552 (a.u.) ΔE= 0.0 Kcal/mol

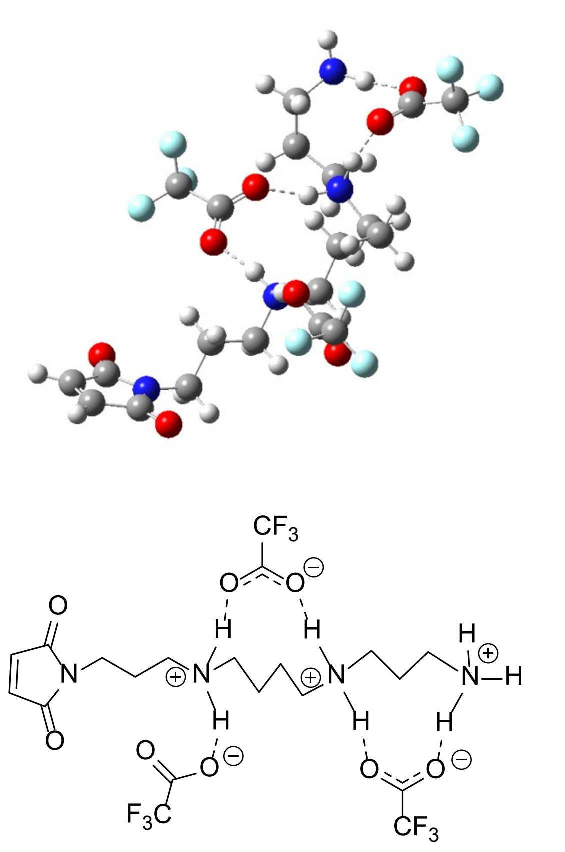

Figure S2-g
E=-2499.142090 (a.u.) ΔE= 2.2 Kcal/mol

As can be seen in the Figures S1-a to S1-d, the favored conformation for X= Cl^- corresponds to figure S1-b. In this conformation, it exists a double bridge N(9)H---Cl---HN(13). For X= $F_3CCO_2^{\ominus}$ (see figures S2-a a S2-g) the situation is different. In this case, the more stable conformation is represented in the figure S2-f. In this situation, it exists a double bridge N(4)H---O=C=O---HN(9) where the anion $F^3CCO_2^{\ominus}$ bonds in a bidentate form.

To build the polycationic nucleus of **4** and **5**, the above-mentioned more stable conformations have been used. In both cases, it has been considered the CIS and TRANS disposition of the side chains.

Figure S3. Conformation diagrams of perylene compound 4. The compound is shown at their calculated lowest energy conformation (ball and stick rendering).

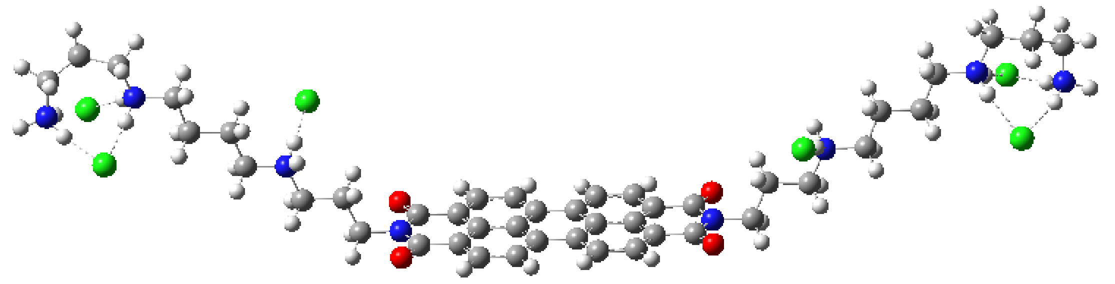

Figure S3-a
E= -5214.689104 (a.u.) ΔE= 0.0 Kcal/mol

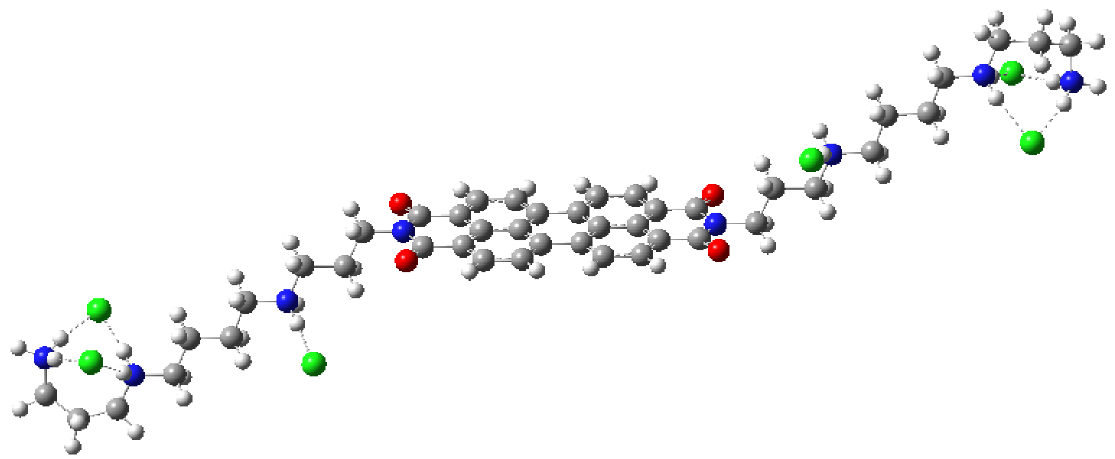

Figure S3-b
E= -5214.689043 (a.u.) ΔE= 0.0 Kcal/mol

Both dispositions (cis and trans) possess the same energy and there is a fast conformational equilibrium between them. Also, rotations around the bonds N-C and C-C of the side chains can be produced.

Figure S4. Conformation diagrams of perylene compound 5. The compound is shown at their calculated lowest energy conformation (ball and stick rendering).

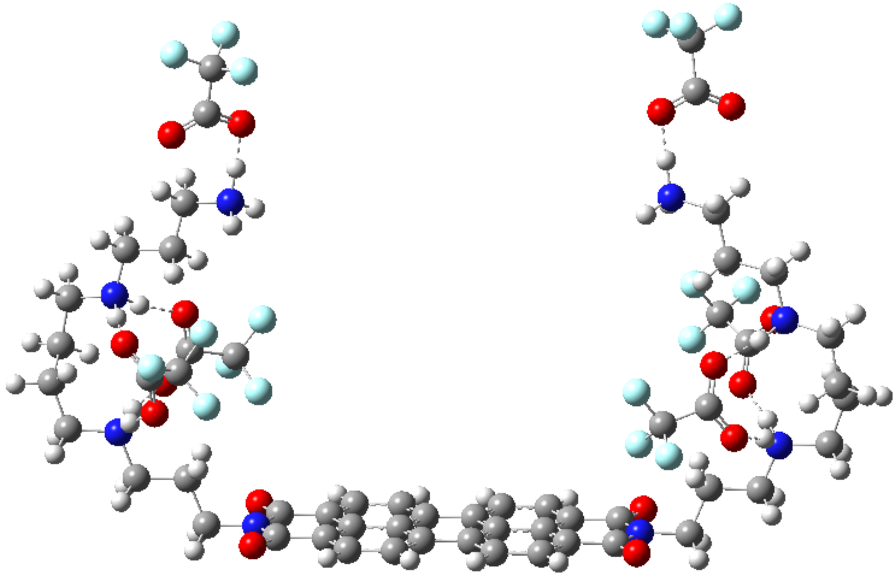

Figure S4-a
E= -5610.555693 (a.u.) ΔE= 0.0 Kcal/mol

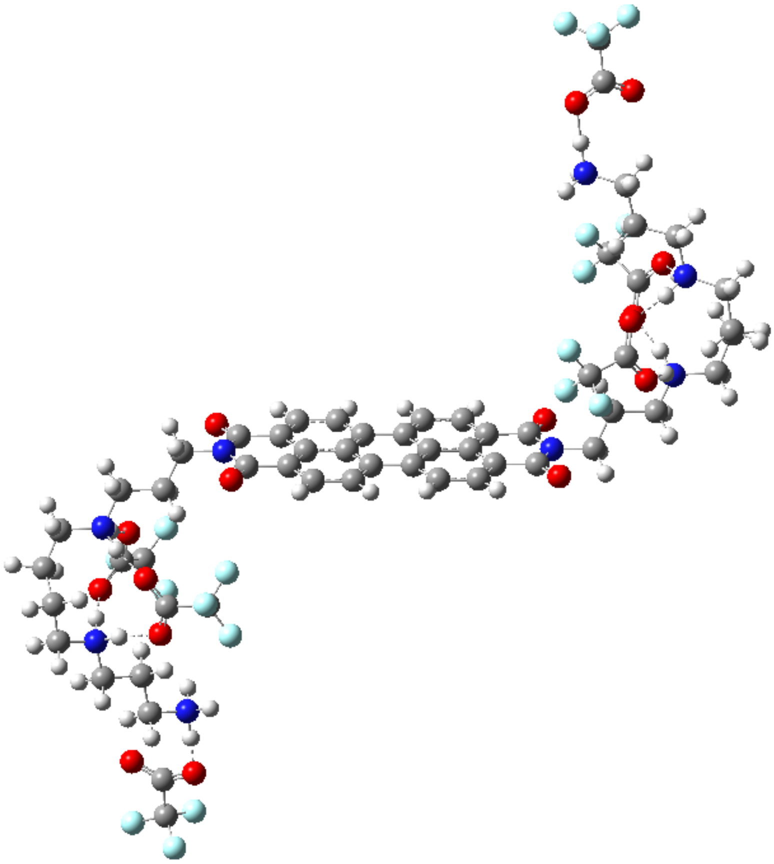

Figure S4-b
E= -5610.555594 (a.u.) ΔE= 0.0 Kcal/mol

In this case, also both dispositions (cis and trans) possess the same energy and a fast conformational equilibrium can be produced as well as rotations around the bonds N-C and C-C of the side chains.

CHELPG charges

-Hexa-trifluoroacetate: -4.22 in the trifluoroacetates and therefore +4.22 in the nucleus (cis and trans).

-Hexa-chloride: -3.99 in the chlorides and therefore +3.99 in the nucleus (cis and trans).

Surfaces of electron density

Compound **4 CIS**

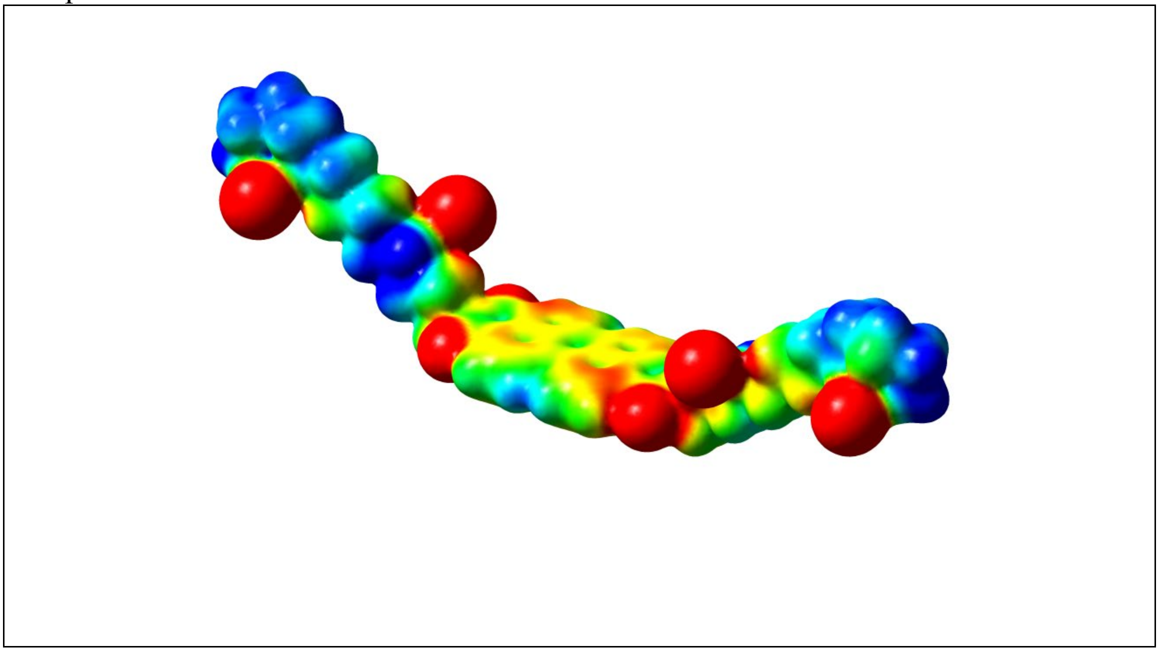

Compound **4 TRANS**

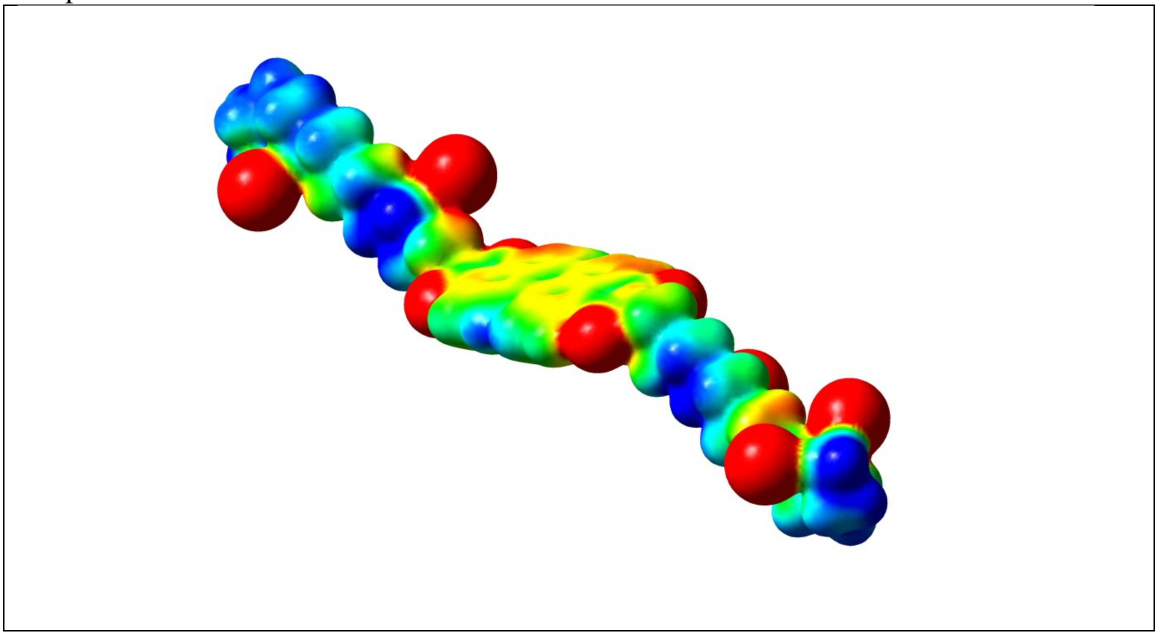

Compound **5 CIS**

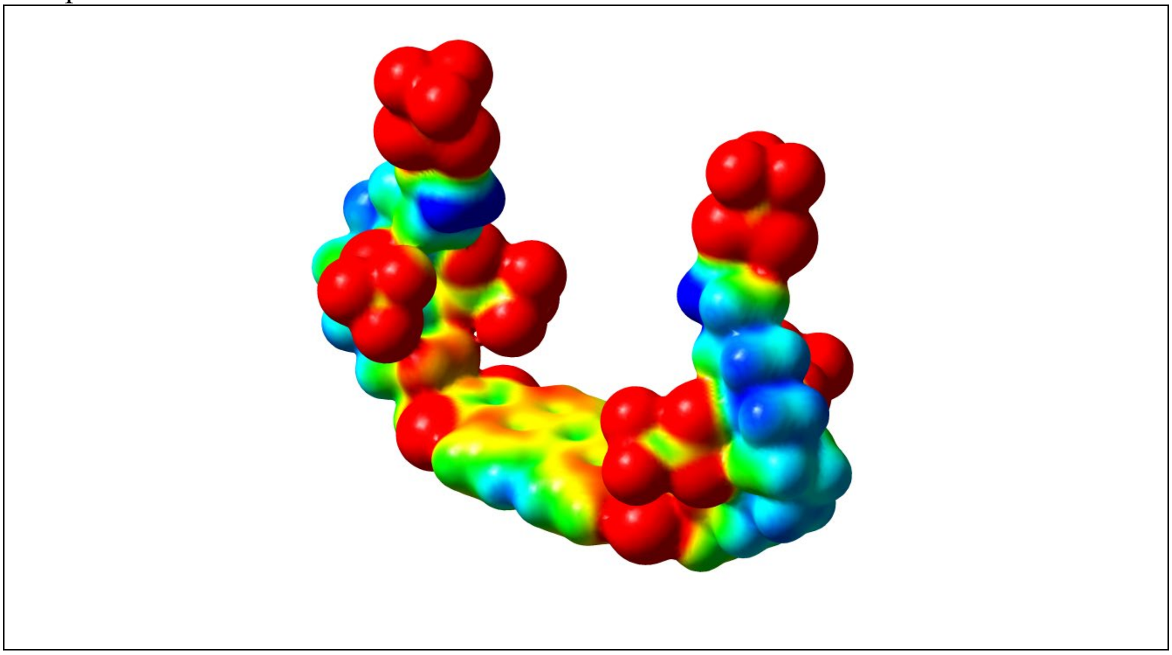

Compound **5 TRANS**

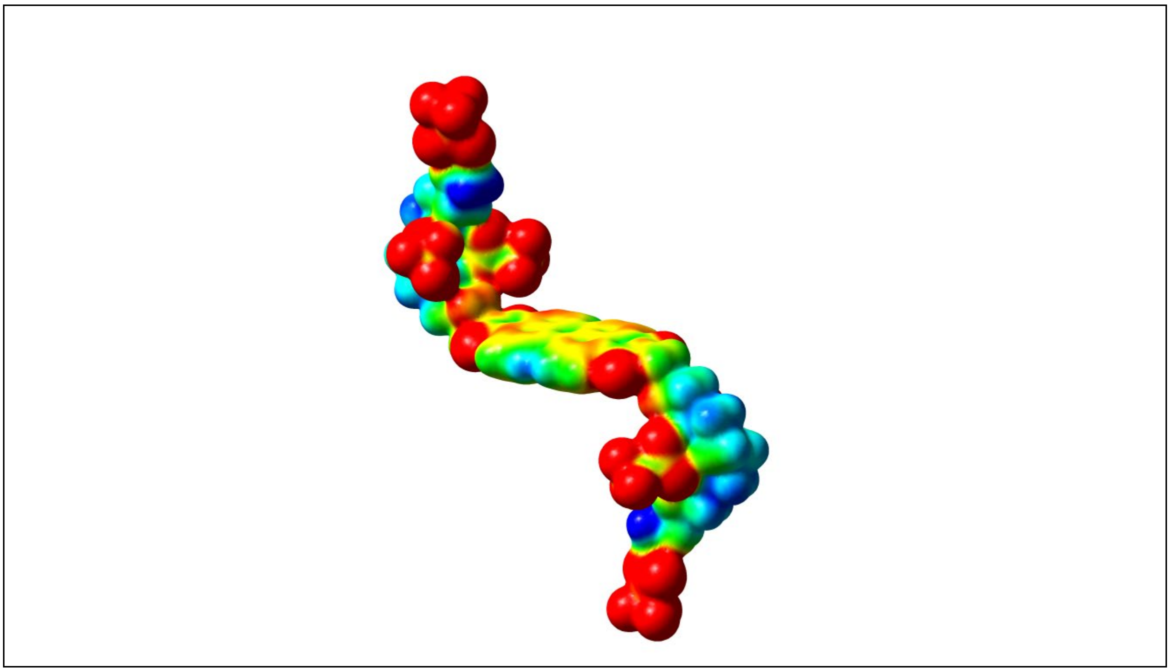

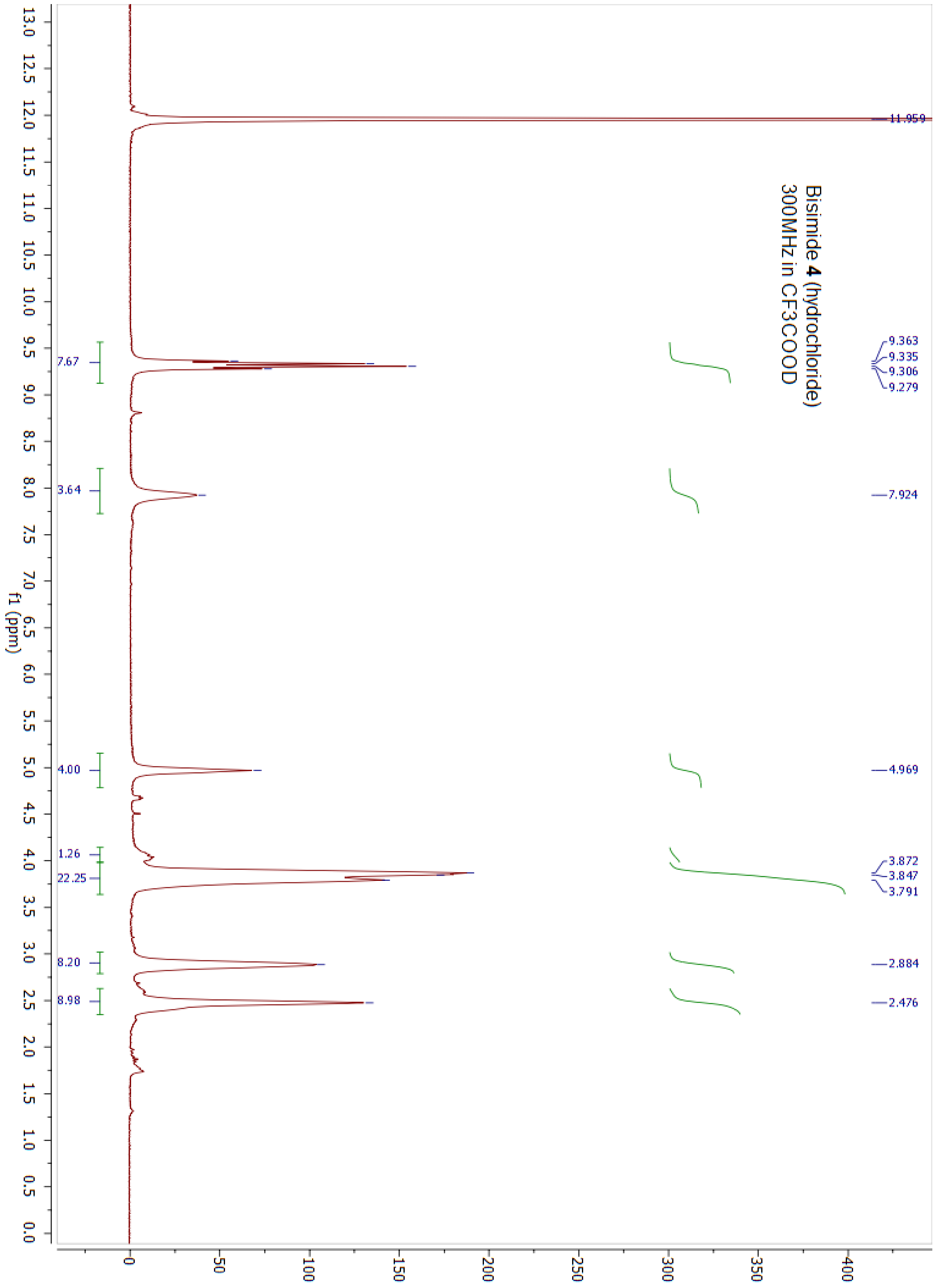
Bisimide 4 (hydrochloride)
300MHz in CF3COOD
11.959
9.363
9.335
9.306
9.279
7.924
4.969
3.872
3.847
3.791
2.884
2.476
7.67
3.64
4.00
1.26
22.25
8.20
8.98
f1 (ppm)

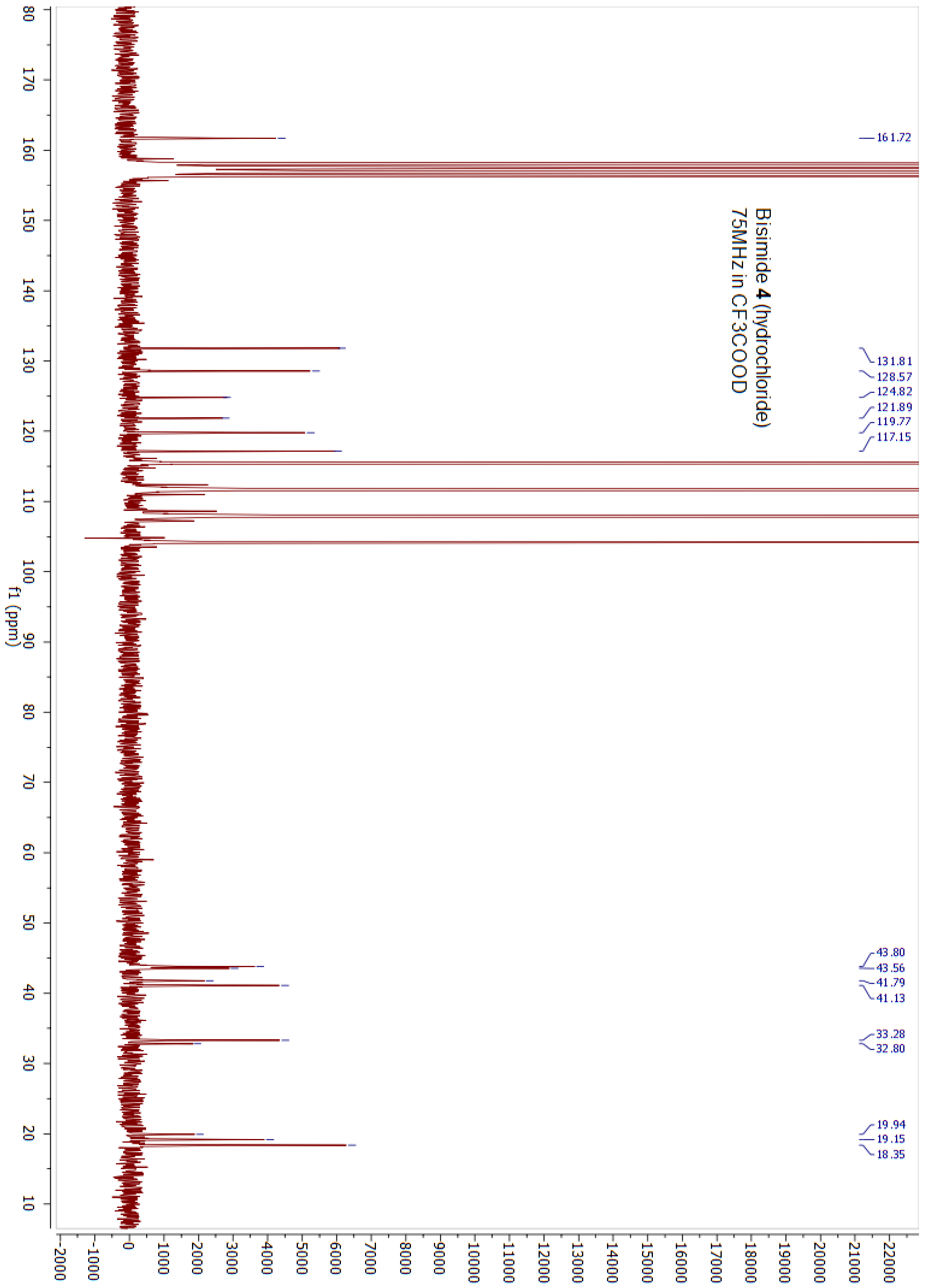
Bisimide 4 (hydrochloride)
75MHz in CF3COOD
161.72
131.81
128.57
124.82
121.89
119.77
117.15
43.80
43.56
41.79
41.13
33.28
32.80
19.94
19.15
18.35
f1 (ppm)

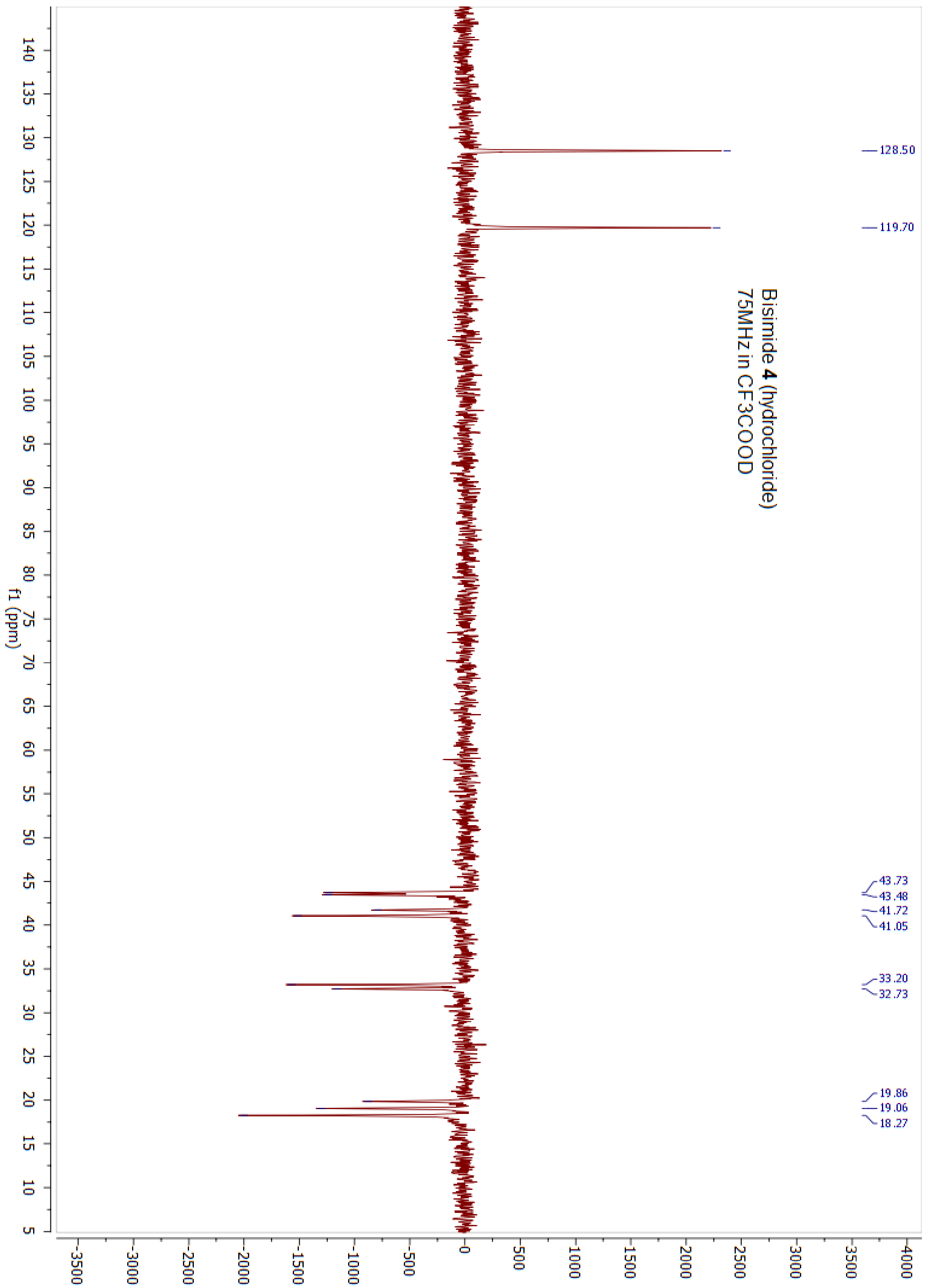
Bisimide 4 (hydrochloride)
75MHz in CF3COOD
128.50
119.70
43.73
43.48
41.72
41.05
33.20
32.73
19.86
19.06
18.27
f1 (ppm)

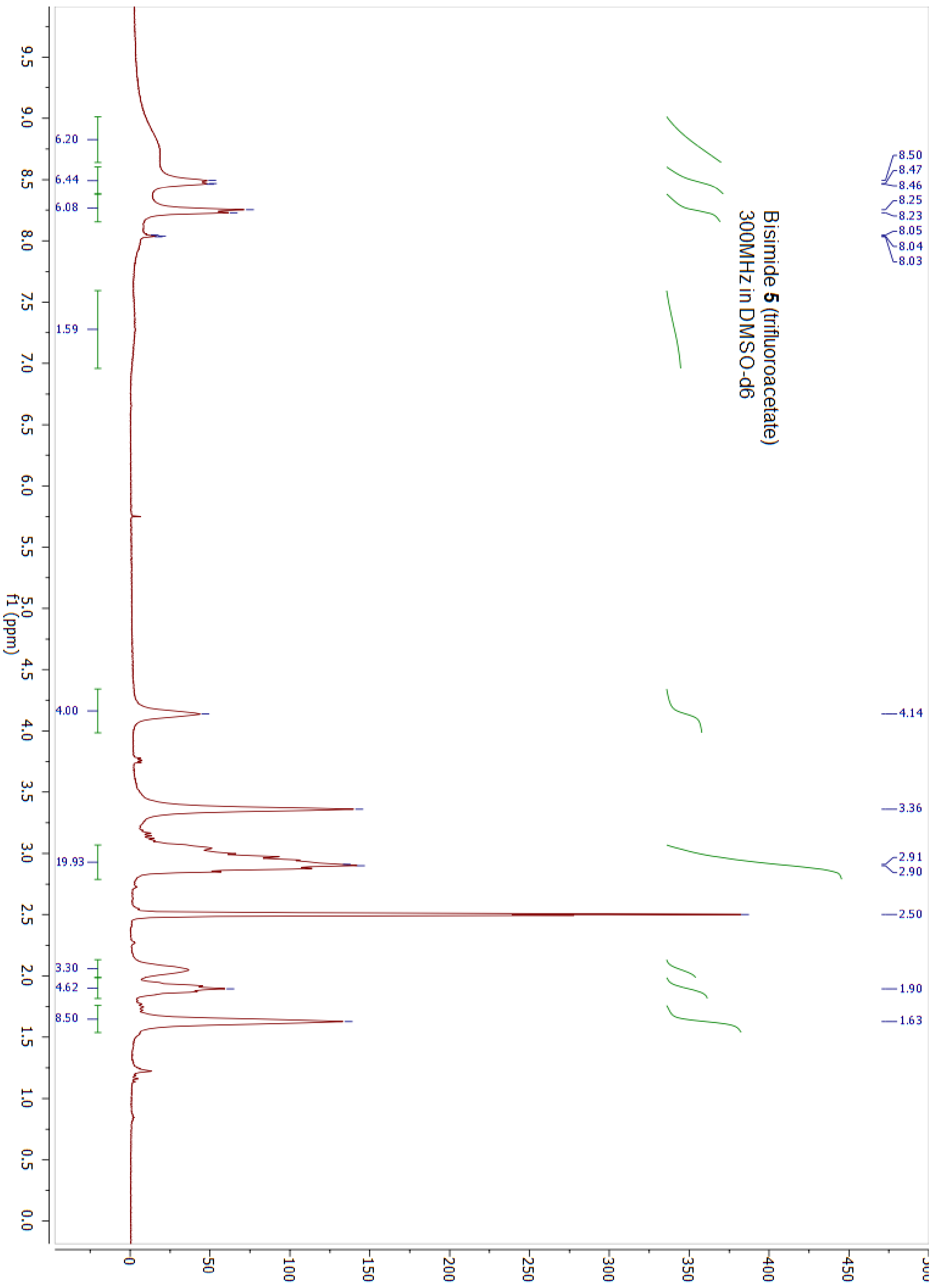
Bisimide 5 (trifluoroacetate)
300MHz in DMSO-d6
8.50
8.47
8.46
8.25
8.23
8.05
8.04
8.03
4.14
3.36
2.91
2.90
2.50
1.90
1.63
6.20
6.44
6.08
1.59
4.00
19.93
3.30
4.62
8.50
f1 (ppm)

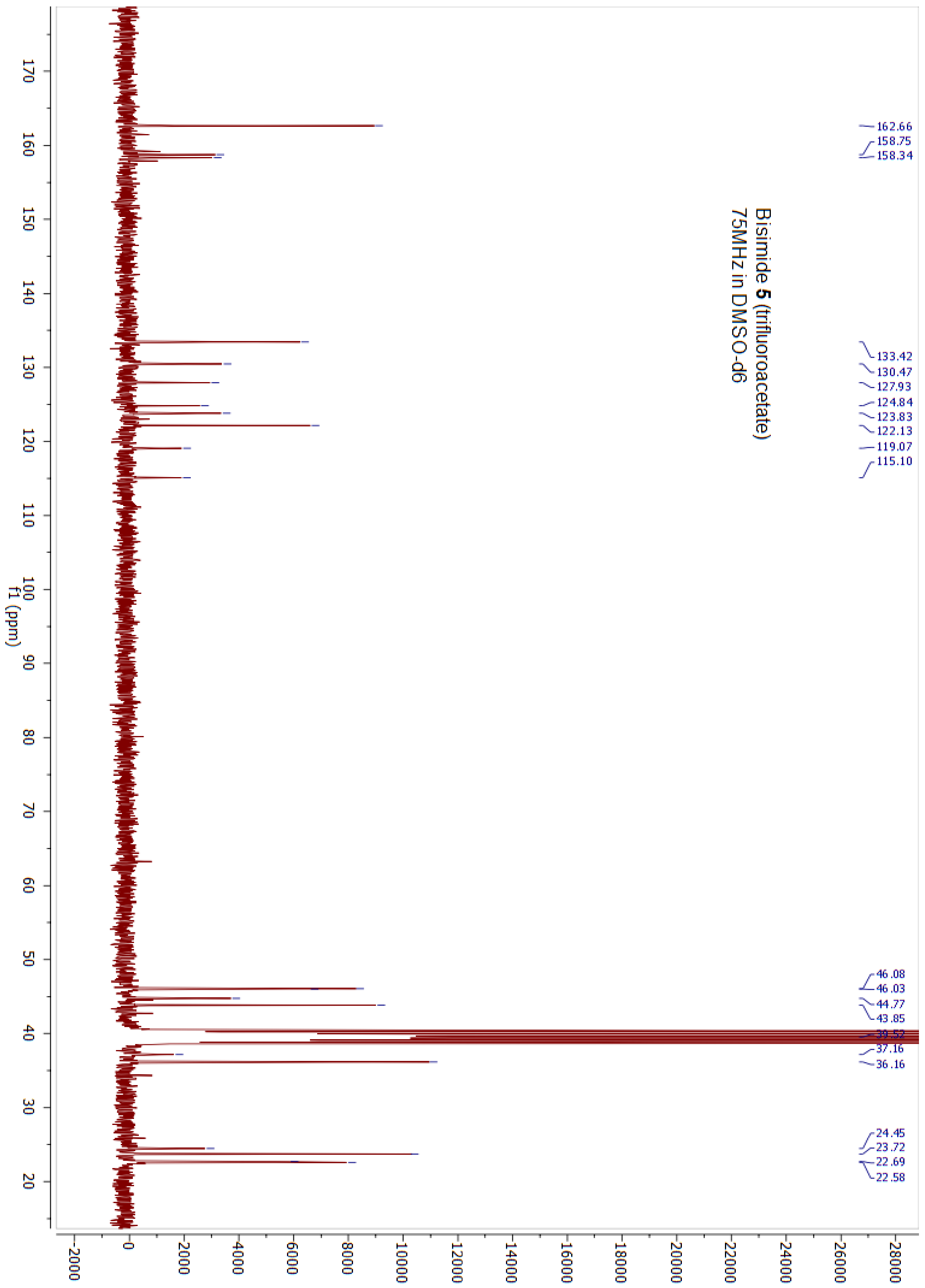
Bisimide **5** (trifluoroacetate)
75MHz in DMSO-d6
162.66
158.75
158.34
133.42
130.47
127.93
124.84
123.83
122.13
119.07
115.10
46.08
46.03
44.77
43.85
37.16
36.16
24.45
23.72
22.69
22.58
f1 (ppm)

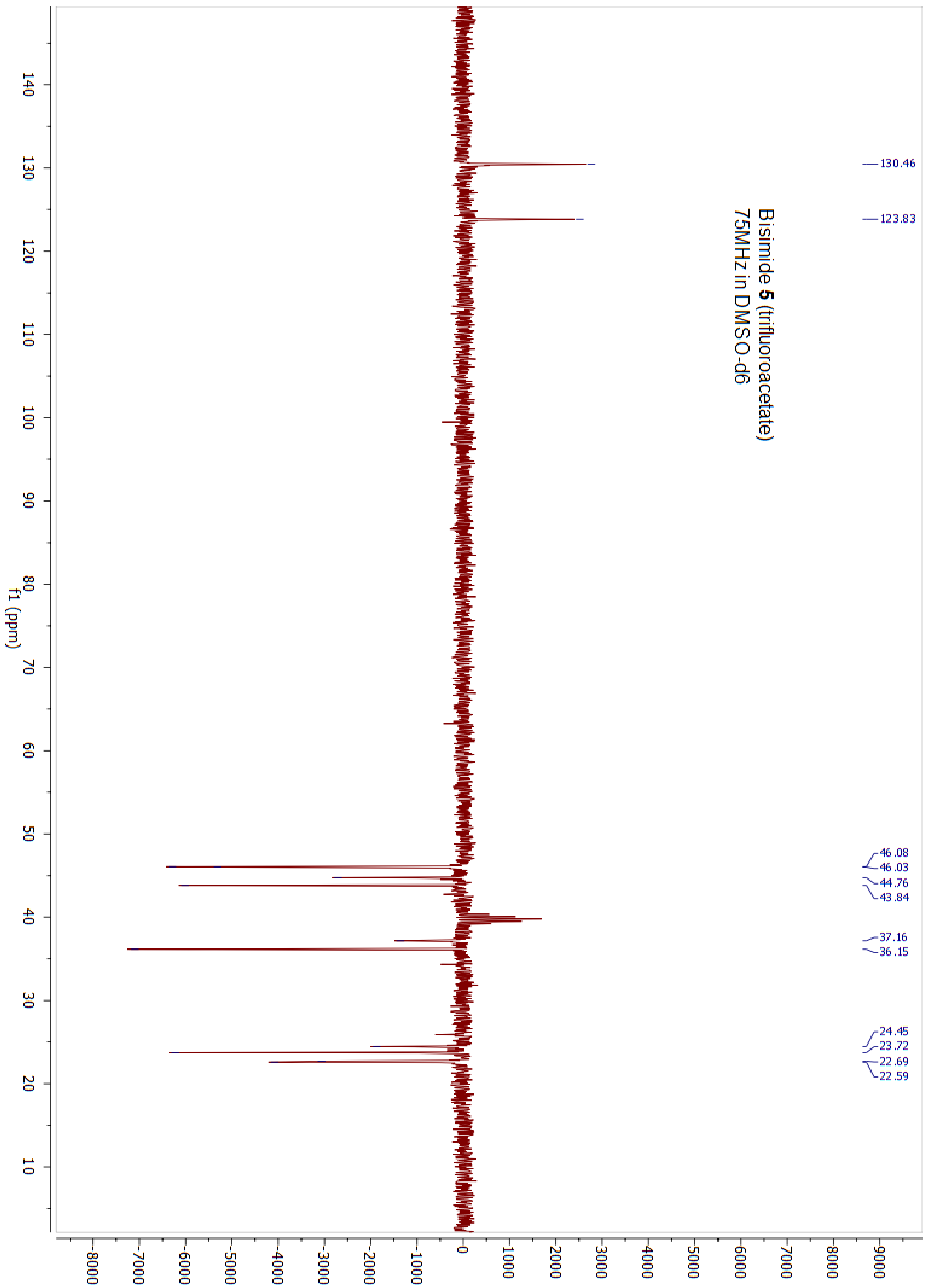

Bisimide 5 (trifluoroacetate)
75MHz in DMSO-d6
130.46
123.83
46.08
46.03
44.76
43.84
37.16
36.15
24.45
23.72
22.69
22.59
f1 (ppm)
140
130
120
110
100
90
80
70
60
50
40
30
20
10
-8000
-7000
-6000
-5000
-4000
-3000
-2000
-1000
0
1000
2000
3000
4000
5000
6000
7000
8000
9000
